# Quantitative Evaluation of the Influence of Posterior Malleolus Fracture and Fixation on the Rotational Stability of the Ankle

**DOI:** 10.1007/s43465-023-00951-1

**Published:** 2023-07-17

**Authors:** Yongqi Li, Haichao Zhou, Jiang Xia, Bing Li, Youguang Zhao, Wenbao He, Zhendong Li, Yunfeng Yang

**Affiliations:** 1 Department of Orthopedics, Tongji Hospital, School of Medicine, Tongji University, Shanghai, 200065 China; 2 Department of Orthopedics, Karamay Central Hospital, Karamay, 834000 China

**Keywords:** Posterior malleolus fracture, Surgical fixation, Syndesmosis, Rotational stability

## Abstract

**Background:**

This study aimed to analyze quantitative correlation between the posterior malleolus fracture and fixation and the rotational stability of the ankle and to explore supplementary surgical indications for posterior malleolus fracture.

**Methods:**

Twenty fresh frozen cadaver specimens were selected and dissected. Based on the tibial insertion of the ligament complex, the model for the supination external rotation stage 3 ankle fracture with a posterior malleolar fragment and syndesmosis diastasis was created. The area threshold of the posterior tibial insertion of posterior malleolus fracture was biomechanically assessed and the difference of the antirotating ability stiffness of the ankle between simple posterior malleolus fixation and simple syndesmotic fixation was analyzed statistically.

**Results:**

The tibial insertion of posterior inferior tibiofibular ligament and inferior transverse tibiofibular ligament complex was relatively broad, and its width decreased as the distance from the joint line increased. Biomechanical analysis showed that: the threshold of posterior area of posterior malleolus fracture was 1/4S; posterior malleolus fixation provided better rotational stability than syndesmotic fixation (*P* *< *0.01).

**Conclusion:**

The surgical indications for posterior malleolus fracture should consider simultaneously the restoration of the axial and rotational stability of the ankle. Simple posterior malleolus fracture fixation is recommended when the syndesmosis is unstable and the area ratio of posterior tibial insertion of posterior malleolus fracture is greater than or equal to 1/4. Syndesmotic fixation is proposed to restore and maintain the rotational stability of the ankle when the syndesmosis is unstable and the area ratio is less than 1/4. Regardless of the area ratio, the surgical indication only depends on the impact of the posterior malleolus fracture on the axial stability of tibiotalar joint, the involved articular surface area, and the displacement degree of posterior malleolus fragment, when the syndesmosis is stable.

## Background

The surgical indications for posterior malleolus fracture have long been controversial [1–4]. Previous related studies have mainly focused on the influence of the posterior malleolus fracture on the axial stability of the tibiotalar joint, such as the posterior talar subluxation, involved articular surface area, and displacement degree of posterior malleolus fragment, and 25% of the articular involvement has been considered as the threshold for surgical treatment [5–7]. The presence of surgical indication for posterior malleolus fracture with small articular involvement is unknown. According to the literature, some posterior malleolus fractures that involved less than 25% of the articular surface still require surgical fixation, but the specific fracture type remains ambiguous [8–11].

The influence of posterior malleolus fracture on the stability of the ankle includes the posterior axial stability and posterolateral rotational stability [10, 12]. Whether posterior malleolus fracture with small articular involvement (such as less than 25%) requires surgical fixation is mainly depended on its impact on the rotational stability of the ankle. Based on the cadaver specimen model, in the current study, the influence of the posterior malleolus fracture with different area ratios of the posterior tibial insertion on the rotational stability of the ankle was biomechanically analyzed to further explore the surgical indications of the reduction and fixation of the posterior malleolus fracture. The results had important theoretical significance for the posterior malleolus fracture involving a small articular surface and are regarded as necessary supplements to previous surgical indications.

## Method

### Experimental Specimen

**Fig. 1:**
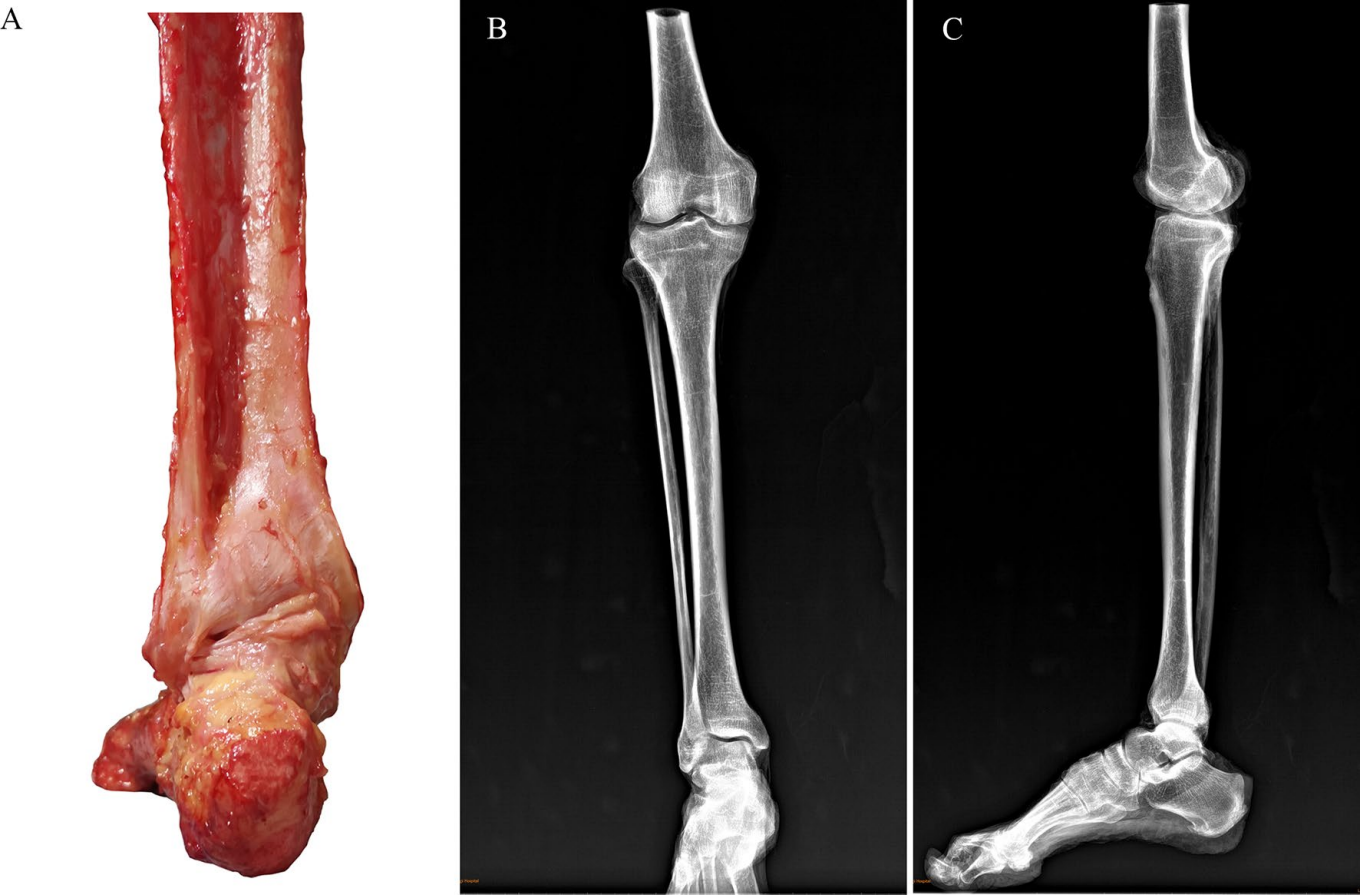
Fresh cadaver specimen. **A** The soft tissue structure around the knee joint and calf was removed, but the deltoid ligament, the lateral collateral ligament, the syndesmotic ligaments, the interosseous membrane, and the ankle joint capsule were preserved; **B** X-rays were performed to rule out abnormal bone changes

Twenty fresh frozen cadaver specimens were selected. The cadavers were patients with average age of 71.2 (range: 58–86) years. Each specimen was amputated at 10–15 cm above the knee and was kept in a low-temperature freezer at − 20 ℃. All specimens were normal in appearance and had no pathological changes, such as foot and ankle deformities and injuries and contractures of the muscle, tendon, and ligament. The specimens were X-rayed to rule out abnormal changes in the bone. The specimens were taken out of the freezer 12 h before the experiment and were naturally thawed at room temperature. The skin, muscles, tendons, nerves, and blood vessels around the knees and calf were removed, but the deltoid ligament, lateral collateral ligament, syndesmotic ligaments, interosseous membrane, and ankle joint capsule were preserved (Fig. 1). The knee joint was fixed at 0° extension position with two 6.5 mm cancellous bone screws, and the subtalar joint was fixed in a neutral position with two 3.5 mm cortical screws. The study was approved by the internal review board of the authors’ hospital.

### Dissection of the Cadaver and Measurement of the Extent of the Tibial Insertion of the PITFL and ITTFL Complex

**Fig. 2:**
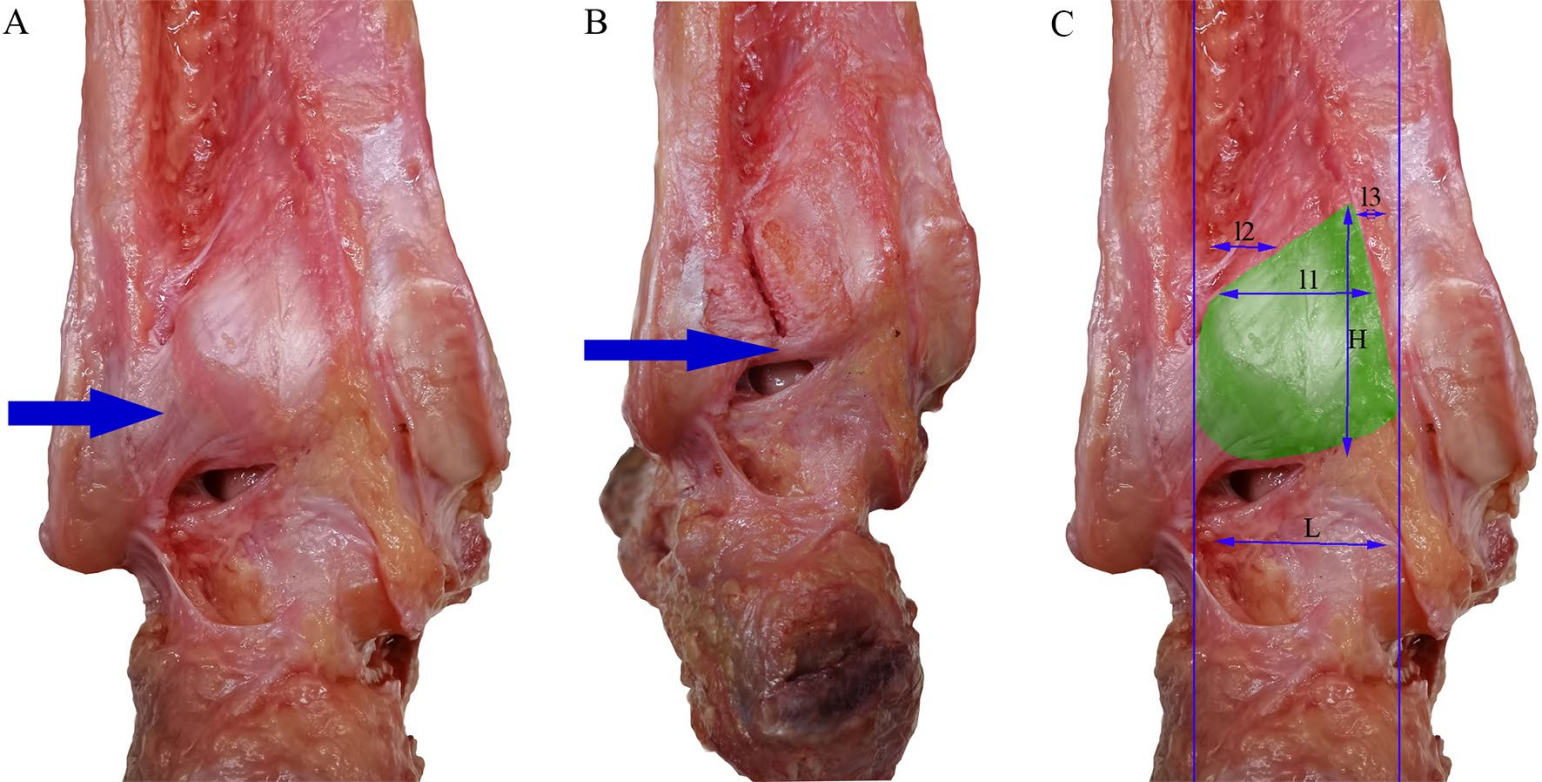
Dissection and measurement of the tibial insertion of the posterior inferior tibiofibular ligament (PITFL) and inferior transverse tibiofibular ligament (ITTFL) complex. **A** PITFL (arrow); **B** ITTFL (arrow); and **C** measurement of the tibial insertion of PITFL and ITTFL complex

The tibial insertion of the PITFL and ITTFL complex was dissected and exposed. A digital caliper (calibrated to 0.02 mm) was used to directly measure the following distances: between the PITFL highest point and the joint line (H1); between the ITTFL highest point and the joint line (H2); and between the lateral edge of the malleolar groove and the posterior tibial tubercle line (L). Meanwhile, at different heights from the joint line (10 mm increment), the width of tibial insertion of PITFL and ITTFL complex (l1), the distance between the lateral edge of tibial insertion of PITFL and ITTFL complex and the posterior tibial tubercle line (l2), and the distance between the medial edge of tibial insertion of PITFL and ITTFL complex and the lateral edge of the malleolar groove (l3) were measured (Fig. 2).

### Establishment of the Model for Supination External Rotation Stage 3 Ankle Fracture

In all 20 specimens, the anterior tibiofibular ligament, interosseous ligament, and interosseous membrane (6 cm above the plafond) were sectioned in the same order. Then, an osteotome was used to create a posterior malleolar fragment with different area ratios of the posterior tibial insertion, and the residual ligament complex outside the fragment was further severed. Thus, this process clinically and experimentally led to the syndesmotic instability [12]. Posterior malleolus fragment involved 1/5 of the articular surface of the distal tibia, and the involved areas of the tibial insertion of the PITFL and ITTFL complex were 1/8S (model 1), 1/4S (model 2), 1/2S (model 3), and S (model 4). The component of PITFL and ITTFL complex on the fragment should not be damaged when the posterior malleolar osteotomy was performed. In addition, the fibula was left intact to simulate the rigid fixation of the fibula fracture without introducing an additional variable of fibular fixation.

### Osteotomy of Posterior Malleolus

#### Osteotomy of the Posterior Malleolus Involving 1/5 of the Articular Surface

Based on the Macko [9] method, posterior malleolus osteotomy was performed to create a Haraguchi type I posterior malleolar fracture model. With fluoroscopy, the posterior 1/5 landmark point was first determined according to the length of the line connecting the anterior and posterior margins of the fibular notch on the tibia. The landmark point was connected to the most medial point of the tibial insertion of the PITFL and ITTFL complex. Then, the posterior fragment involved 1/5 of the articular surface of the distal tibia.

#### Osteotomy of the Posterior Malleolus Involving Different Areas of Tibial Insertion of PITFL and ITTFL Complex

##### Principle of Osteotomy

**Fig. 3:**
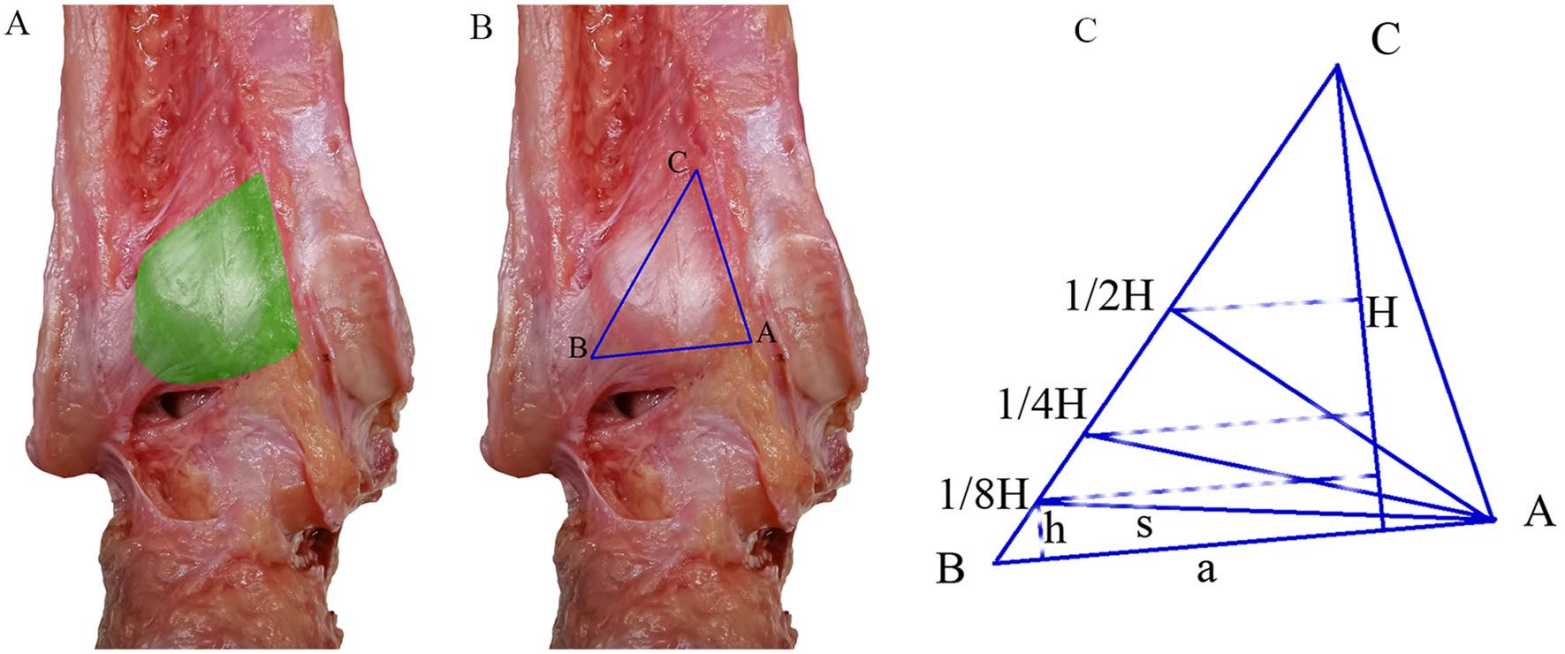
Schematic diagram of the osteotomy of the posterior malleolus fragment involving different areas of the posterior tibial insertion of the PITFL and ITTFL complex. **A** The green shadow showed the tibial insertion of the PITFL and ITTFL complex; **B** the tibial insertion of ligament complex was similar to a triangle with the posterior lip of the tibia as the base (BA) and the highest point **C** of tibial insertion as the apex; **C** schematic diagram of the method of posterior malleolus osteotomy based on tibial insertion of ligament complex. Point B was the lateral edge of posterior tibial tubercle. Point A was the most medial point of the tibial insertion of ligament complex. Point C was the highest point of the tibial insertion of ligament complex, and the letters a, h, and s represented the length, height, and area, respectively, of the posterior malleolus osteotomy fragment on the posterior surface of the tibia

The tibial insertion of the PITFL and ITTFL complex was similar to a triangle with the posterior lip of the tibia as the base (BA) and the highest point (C) of tibial insertion as the apex. If the length of the base and height of the triangle were set to a and H, respectively, its area was S (S = a×H/2). Then, when the length of the base was still a and the height h was equal to H, 1/2H, 1/4H, and 1/8H, the areas of the distal posterior malleolus fragment corresponded to S, 1/2S, 1/4S and 1/8S (Fig. 3).

##### Method of Osteotomy

**Fig. 4:**
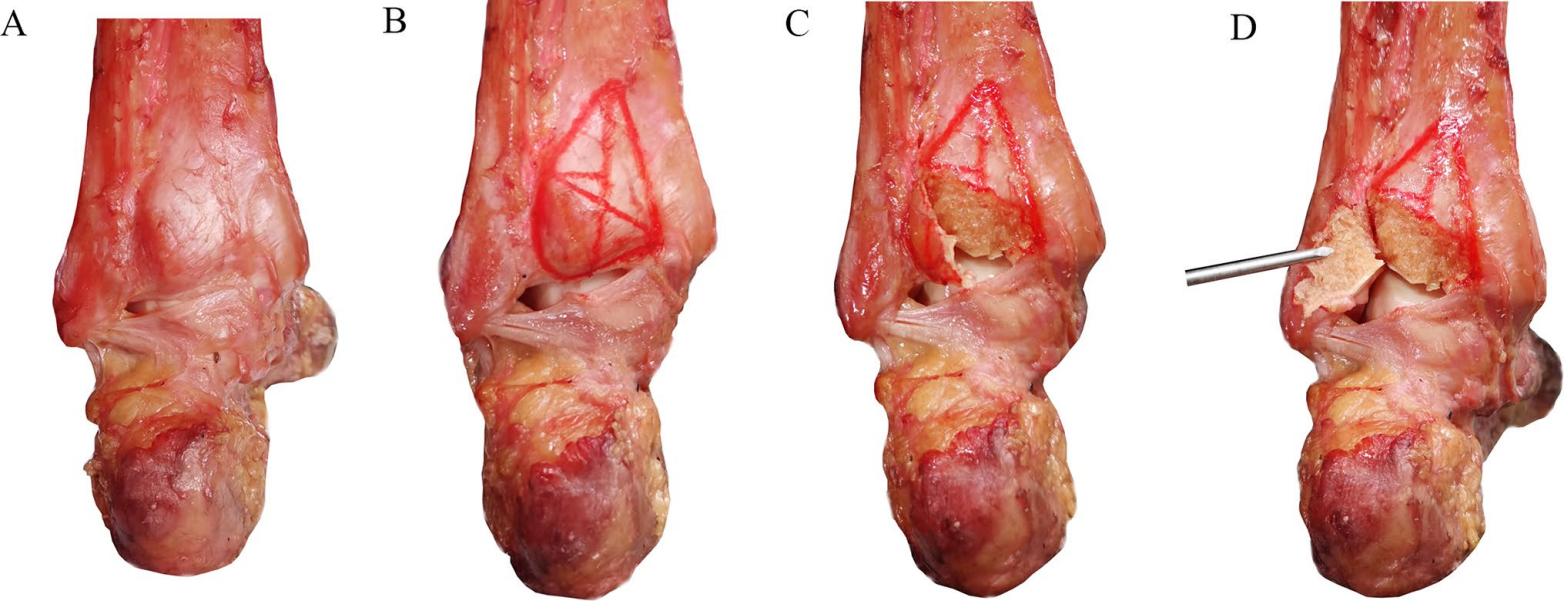
An example of the posterior malleolus osteotomy with fragment involving 1/2S of the tibial insertion of PITFL and ITTFL complex on the posterior surface of the tibia

First, on the posterior surface of the tibia, the triangle of tibial insertion of PITFL and ITTFL complex was marked with a marker. Second, H was measured and points 1/2H, 1/4H, and 1/8H) were marked. Then, the horizontal line passing through the point intersected the lateral side of the triangle. Finally, the intersection was connected to the most medial point of the tibial insertion of PITFL and ITTFL complex (A). Then, the areas of the new triangles below were 1/2S, 1/4S, and 1/8S (Figs. 3 and 4).

### Analysis of the Area Threshold of the Posterior Tibial Insertion of Posterior Malleolus Fracture (SF)

#### Experimental Scheme

**Fig. 5:**
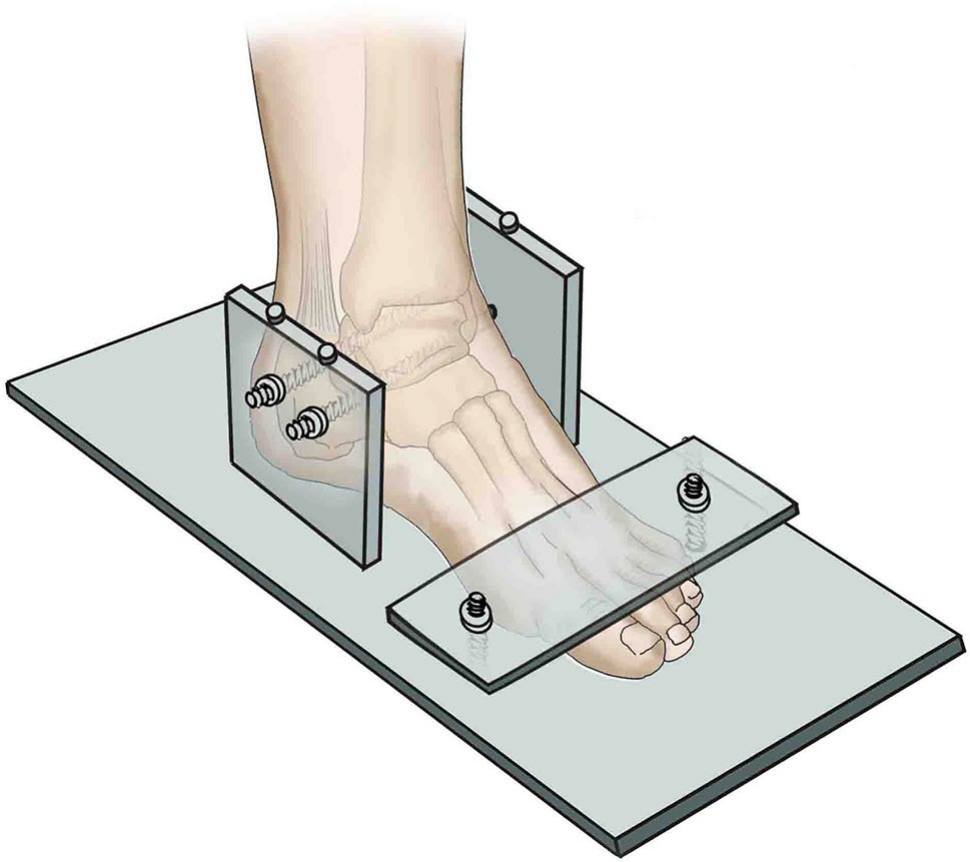
Fixation of the posterior malleolus fracture model. The foot was secured to the base plate with two parallel 4.0 mm Denham pins passing through the calcaneum. A metal plate was placed over the dorsal forefoot to control the rotational and vertical movements

**Fig. 6:**
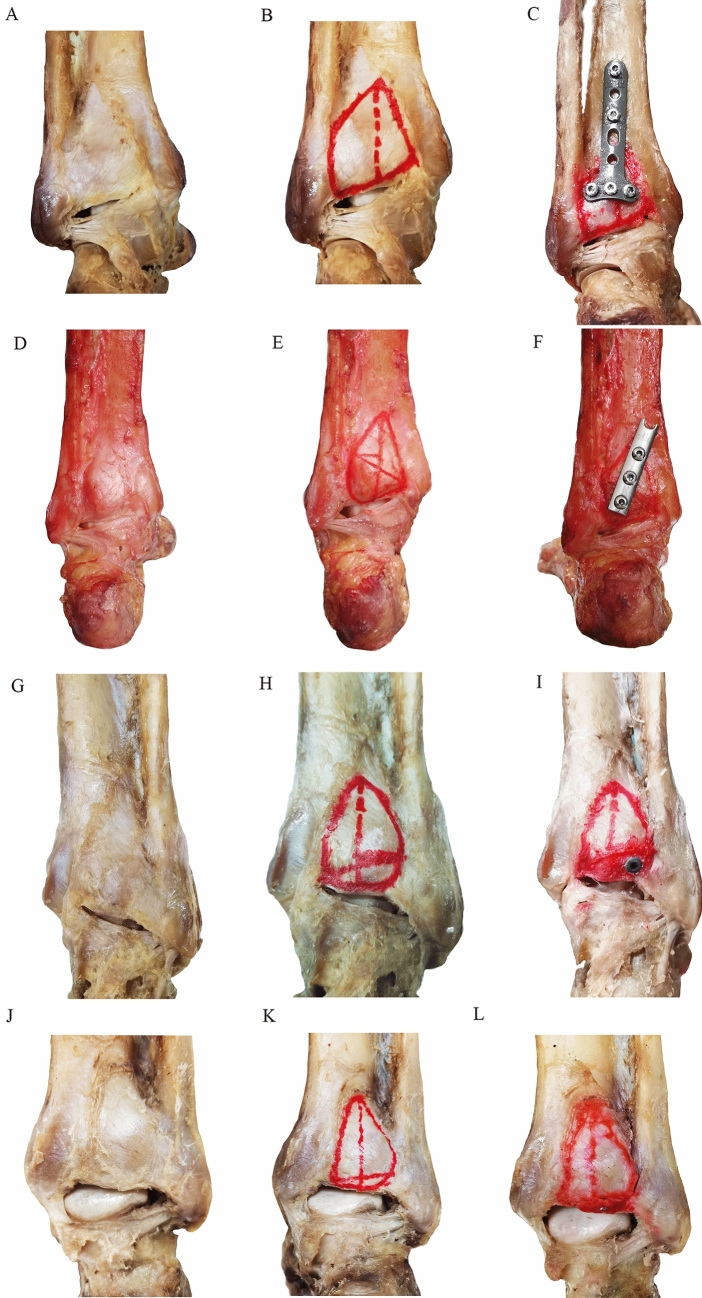
Osteotomy and fixation of posterior malleolus involving different areas of tibial insertion of the PITFL and ITTFL complex. (**A**–**C**) Model 4 (posterior area of posterior malleolus: (S) and buttress plate fixation; (**D**–**F**) Model 3 (1/2S) and buttress plate fixation; (**G**–**I**) Model 2 (1/4S) and screw fixation (**J**–**L**) Model 1 (1/8S) and screw fixation

The femur of the specimen was fixed to the proximal end of the DDL20 electronic testing machine. The foot was secured to the base plate with two parallel 4.0 mm Denham pins passing through the calcaneum. A metal plate was placed over the dorsal forefoot to control the rotational and vertical movements. From models 1 to 4, the posterior malleolus fractures were reduced and fixed sequentially and loaded axially and rotationally. Then, the syndesmosis stability was assessed. If simple posterior malleolus fixation could not maintain the syndesmosis stability, the syndesmotic screw fixation would be increased. The first model in which the simple posterior malleolus fixation could maintain syndesmosis stability was recorded as MS. The involved tibial insertion area of the posterior malleolus fracture corresponding to the model MS was recorded as the threshold SF (Figs. 5 and 6).

#### Reduction and Fixation of the Posterior Malleolus Fracture Model

The posterior malleolus fracture was reduced anatomically under direct vision. Using the standard AO technique, model 1 (1/8S) and model 2 (1/4S) were stabilized with a 3.5 mm or 2.7 mm postero-anterior cortical screw, while model 3 (1/2S) and model 4 (S) were stabilized with a buttress plate. In syndesmotic fixation, one 3.5 mm tricortical screw was placed from lateral to medial, angled 30° anteriorly at 2 cm above and parallel to the plafond.

#### Loading Method

Each specimen was mounted in the neutral position of the ankle and loaded axially and rotationally [10]: first, the specimen was axially loaded with 600 N at 10 mm/min. Then, with the stable axial load, the ankle was externally rotated to 5 Nm at a torsion rate of 1°/s. The tibiofibular clear space and the maximum external rotation of the foot were measured after the load was maintained for 30 s.

#### Definition of the Fixation Failure of Posterior Malleolus Fracture to Maintain the Syndesmosis Stability

The disruption of the ligament complex component or avulsion fracture on the posterior malleolus fragment was visualized. The tibiofibular clear space increased by more than 2 mm. The maximum external rotation of the foot was restored to the level before the posterior malleolus fixation.

#### Measurement of the Tibiofibular Clear Space and the Maximum External Rotation of the Foot

The tibiofibular clear space was measured according to the direct measurement method of Gosselin-Papadopoulos [11]. Before the establishment of the posterior malleolus osteotomy model, according to the above method, the specimen was fixed in the neutral position and loaded on the electronic testing machine. Two smooth 0.8 mm Kirschner wires were inserted into the posterolateral edge of the tibia and posteromedial edge of the fibula from the posterior to anterior to act as reference points for the anatomical measurements. The wires were located 1 cm above the tibial plafond and parallel to the plantar surface of the foot. On the posterior surface of the tibia, the distance between the bases of the two Kirschner wires was directly measured using a digital caliper and served as the tibiofibular clear space when the posterior malleolus and syndesmosis were intact. In this measurement, the 3-plane motion of the syndesmosis was considered, and a single value similar to the intraoperative assessment of the posterior syndesmosis was created. After the measurement, the Kirschner wire of the tibia was further partially penetrated forward. However, part of this wire was still retained in the tibia, and the depth standard was established such that the Kirschner wire did not affect the posterior malleolus osteotomy. After osteotomy and fixation of the posterior malleolus, the specimen was fixed and loaded according to the above method again. The wire was retrogradely passed through the tibial posterior surface or the fragment from anterior to posterior. The distance between the bases of the two Kirschner wires was measured again as the tibiofibular clear space after the posterior malleolus fixation. The difference between the two measurement values was the increase in the tibiofibular clear space. The external rotation of the foot was directly measured and displayed by the testing machine.

### Comparison of the Antirotation Ability of the Ankle Stiffness Between Simple Posterior Malleolus Fixation and Simple Syndesmotic Fixation

#### Experimental Scheme

Among the different cadaver specimen models, the models in which the simple posterior malleolus fixation could maintain the syndesmosis stability were selected. Simple posterior malleolus fixation and simple syndesmotic fixation were performed separately. Then, each specimen was loaded with the external rotation of the foot. The maximum external rotation angle of the foot was measured, and stiffness was calculated as stiffness=torque/maximum external rotation angle.

#### Loading Method

The method of specimen fixation was the same as before. Initially, a static axial load of 150 N was applied to ensure the joint contact. The position was maintained, and the torsional load of external rotation was performed at 1°/s. When the torque reached 4 Nm, the maximum external rotation angle of the foot was measured, and the stiffness was calculated [12].

### Statistical Analysis

SPSS 25 software was used to analyze the data. Tibiofibular clear space (TFS) increase and stiffness of the ankle followed a normal distribution (Kolmogorov–Smirnov test), and were presented as mean and standard deviation (x ± s). Independent sample t test was used for comparison of TFS increase between different posterior malleolus fracture models. Paired sample t test was used for comparison of the stiffness between two fixation methods. The significance level was set to *p* < 0.05.

## Results

The PITFL and ITTFL were present in all specimens and could be distinguished clearly. The PITFL tibial insertion was relatively broad. The PITFL was attached to the posterolateral tibia and the proximal and superficial surfaces of the ITTFL, and this ligament was blended with the tibialis posterior tendon sheath medially. The average distance between the highest point of the tibial insertion and the articular line was 45.2 ± 5.6 mm. The ITTFL was attached to the posterior distal tibia and deep surfaces of the PITFL, and this ligament was also blended with the tibialis posterior tendon sheath medially. The average distance between the highest point of the tibial insertion and the articular line was 5.5 ± 1.0 mm. The width of the tibial insertion of the PITFL and ITTFL complex (l1) decreased as the distance from the joint line increased. The specific measurement values are shown in Table 1.

**Table 1:** Measurement of the extent of the tibial insertion of the PITFL and ITTFL complex

	Measurement value (mm)		l1 (mm)	l2 (mm)	l3 (mm)
L	28.5 ± 4.2	Height from the joint line			
H1	45.2 ± 5.6	10 mm	26.8 ± 3.2	0 ± 0.1	2.0 ± 0.6
H2	5.5 ± 1.0	20 mm	23.1 ± 2.8	1.1 ± 0.3	4.7 ± 1.1
		30 mm	18.2 ± 2.5	4.6 ± 1.8	6.1 ± 1.6
		40 mm	8.5±1.3	12.2 ± 2.3	8.1 ± 2.3

L: the distance between the lateral edge of the malleolar groove and the posterior tibial tubercle line

H1: the distance between the PITFL highest point and the joint line

H2: the distance between the ITTFL highest point and the joint line

l1: the width of tibial insertion of PITFL and ITTFL complex

l2: the distance between the lateral edge of tibial insertion of PITFL and ITTFL complex and the posterior tibial tubercle line

l3: the distance between the medial edge of tibial insertion of PITFL and ITTFL complex and the lateral edge of the malleolar groove. L= l1+ l2+ l3

The method of posterior malleolus osteotomy and model establishment proposed in this study were feasible. In the current loading scheme, from model 1 (1/8S) to model 4 (S), no macroscopic failure was noted to maintain the syndesmosis stability after the posterior malleolus fixation. Disruption of the PITFL component or avulsion fracture on the posterior malleolus fragment was observed. The maximum external rotation of the foot was restored to the level before the posterior malleolus fixation. However, the measured value showed a difference in the degree of the increase of the tibiofibular clear space. Only in model 1 (1/8S), the increase in the tibiofibular clear space was greater than 2 mm (2.3±0.3 mm). In addition, the difference between model 1 (1/8S) and model 2 (1/4S) was statistically significant (*P < *0.01). Model 1 (1/8S) was regarded as a failure to maintain the syndesmosis stability with the posterior malleolus fixation. Model 2 (1/4S) was considered as the first group of models that could maintain the syndesmosis stability with simple posterior malleolus fixation, so that the threshold of posterior area of posterior malleolus fracture (SF) was 1/4S (Table 2).

**Table 2:** Comparison of tibiofibular clear space (TFS) increase between different posterior malleolus fracture models

Osteotomy area	TFS (mm) (intact)	TFS (mm) (after posterior malleolus fixation)	TFS increase (mm)	*P*
S(*n*= 5)	3.3 ± 0.2	4.1 ± 0.4	0.8 ± 0.4	–
1/2S(*n *= 5)	3.8 ± 0.3	4.6 ± 0.4	0.8 ± 0.6	–
1/4S(*n*= 5)	3.4 ± 0.3	4.7 ± 0.2	1.1 ± 0.3	< 0.01^a^
1/8S(*n *= 5)	3.5±0.3	5.7±0.2	2.3±0.3	

^a^Independent sample *t* test was used to compare differences between 1/4S group and 1/8S group

Comparison of the stiffness between the two fixation methods showed that posterior malleolus fixation provided better rotational stability than syndesmotic fixation (*P < *0.01). Compared with the intact specimens, stiffness was restored to 60.9% ± 10.2% after posterior malleolus fixation and to 37.5% ± 7.9% after syndesmosis stabilization (Table 3).

**Table 3:** Comparison of the stiffness between simple posterior malleolus fixation (M) and simple syndesmotic fixation(S)

	Intact stiffness (Nm/°)	Stiffness after fixation (Nm/°)	Percentage of stiffness restoration	*P*
M (*n *= 15)	0.430 ± 0.090	0.264 ± 0.080	60.9% ± 10.2%	< 0.01^a^
S (*n *= 15)	0.430 ± 0.090	0.164 ± 0.061	37.5% ± 7.9%	

^a^Paired sample *t* test was used to compare differences between groups

Combining the difference in the antirotating ability of the two fixation methods, the threshold of the posterior area of posterior malleolus fracture and the syndesmosis stability, the recommended surgical indications for posterior malleolus fracture are shown in Table 4.

**Table 4:** Recommended surgical indications and treatment option for posterior malleolus fracture

	Posterior area ratio of posterior malleolus fracture	
	≥ 1/4	< 1/4
Syndesmosis stability		
Unstable	Posterior malleolus fixation	Syndesmotic fixation ± posterior malleolus fixation
Stable	The posterior malleolus fixation indications only depended on the impact on the posterior axial stability, the involved articular surface area, and the displacement degree of posterior malleolus fragment	

## Discussion

Surgical indications for posterior malleolus fracture have long been controversial. In many previous studies, fractures involving more than 25% of the articular surface, displacement greater than 2 mm, and the persistent posterior subluxation of the talus have been considered as surgical indications for posterior malleolus fracture [5, 6, 13]. However, further studies have questioned these indications, such as the appropriateness of 25% as the threshold for the surgical treatment of posterior malleolus fracture. Some studies have recently reported that part of the posterior malleolus fractures involving less than 25% of the articular surface still require surgical fixation [8]. However, the specific fracture type that requires surgical treatment remains inconclusive.

The influence of posterior malleolus fracture on the ankle stability includes the posterior axial stability and posterolateral rotational stability [14, 15]. Previous related studies have mainly focused on the influence of the posterior malleolus fracture on the axial stability of the ankle, but the influence on the rotational stability of the ankle was not fully considered. Whether posterior malleolus fracture with small articular involvement (such as less than 25%) requires surgical fixation is mainly focused on its impact on the rotational stability of the ankle, while its impact on the posterior axial stability is relatively small. Therefore, the surgical indications of posterior malleolus fracture based on the influence of posterior malleolus fracture and fixation on the rotational stability of the ankle were further explored in this study. The indications will have positive theoretical significance especially for posterior malleolus fractures involving a small articular surface area and will serve as supplements to previous surgical indications.

### The Influence of Posterior Malleolus Fracture and Fixation on the Rotational Stability of the Ankle

Reduction and fixation of the posterior malleolus fracture significantly improve the syndesmosis function [16, 17] and restore the rotational stability of the ankle. Gardner et al. pointed out that, in patients with ankle fractures involving the posterior malleolus, the PITFL was still retained and attached to the posterior malleolus fragment [12]. The PITFL has been considered as the most important component of the syndesmosis complex that affects the syndesmosis stability. Reduction and fixation of posterior malleolus fracture fully restored the PITFL tension and stabilized the syndesmosis [18] also believed that, compared with syndesmotic fixation, fixation of the posterior malleolus fracture provides better rotational stability; that is, the stiffness is restored to 70% after fixation of the posterior malleolus and to 40% after syndesmotic fixation. Baumbach et al. [18] and Miller et al. [19, 20] pointed out that the reduction and fixation of the posterior malleolus fracture improves the quality of syndesmosis reduction. Miller et al. believed that the reduction and fixation of the posterior malleolus fracture reduced the incidence of the syndesmosis instability. In the current study, for the model that could maintain the syndesmosis stability with simple posterior malleolus fixation, the stiffnesses with simple posterior malleolus fixation and simple syndesmotic fixation were also measured, and paired design schemes were used. The results showed that posterior malleolus fixation provided better rotational stability than syndesmotic fixation (*P* < 0.01). The restored stiffnesses of simple posterior malleolus fixation and simple syndesmotic fixation were 60.9% ± 10.2% and 37.5% ± 7.9%, respectively (Table 3). The conclusion was the same as that in the literature. However, the values of stiffness in this study were slightly different from those in the literature probably due to the difference in the variation in the freshness of the selected specimens.

### Anatomical Basis of the Influence of Posterior Malleolus Fracture and Fixation on the Rotational Stability of the Ankle

To further understand the relationship between the posterior malleolus fracture and the rotational stability of the ankle, the anatomical characteristics of the PITFL and ITTFL attached to the posterior malleolus have been carefully analyzed. Williams et al. [21] believed that the PITFL had superficial and deep layers. The deep one was referred to as the ITTFL. The superficial fibers were broadly attached to the posterolateral tibial tubercle and blended with the posterior tibial cortex medially. Jayatilaka et al. [22]performed anatomical measurement of the tibial insertion of PITFL in ten cadaver specimens, and the anatomical data of the cadaver specimens were correlated with the CT image data of 80 cases of Mason type 2 posterior malleolus fractures. They proposed that the PITFL proximally split into two separate insertions, namely, an oblique insertion and a transverse insertion. Its tibial insertion was broad. The average size of the tibial insertion was 54.9 mm (95% CI 51.8, 58.0) from the joint line and 47.1 mm (95% CI 43.0, 51.2) transversely. Compared with the average size of the posterior malleolar fragments, the PITFL insertion was significantly larger. In the current anatomical study, the PITFL and ITTFL were present in all specimens, and the extent of the tibial insertion was closer to the results of Jayatilaka et al. The PITFL was attached to the posterolateral tibia and the proximal and superficial surfaces of the ITTFL, and its tibial insertion was relatively broad. The ITTFL was attached to the posterior distal tibia. Both ligaments blended with the tibialis posterior tendon sheath medially. The extent of the tibial insertion of the PITFL and ITTFL complex was also measured quantitatively, and anatomical foundation was established for further biomechanical research (Table 1 and Fig. 2).

### The Establishment of Posterior Malleolar Osteotomy Model Based on Posterior Malleolar-Associated Ligaments and Rotational Stability of the Ankle

The posterior malleolar osteotomy and establishment of the specimen models promoted the study of posterior malleolar biomechanics. In the current experiment, a new posterior malleolar osteotomy scheme was proposed based on the tibial insertion of the PITFL and ITTFL complex. The relationship between the posterior malleolus fracture and fixation and ankle rotational stability was expected to be resolved. To the authors’ knowledge, this study is the first to report the posterior malleolus osteotomy based on the posterior tibial insertion of the ligament complex. In addition, this study is the first work to explore the quantitative correlation between the posterior malleolus fracture and fixation and ankle rotational stability and the surgical indications for posterior malleolus fracture based on the ankle rotational stability. The theoretical basis of this osteotomy was that the PITFL and the ITTFL were approximately evenly attached to the posterior tibia to maintain the posterolateral rotational stability of the ankle. Posterior malleolus osteotomy fragment with different area ratios of the tibial insertion of the PITFL and ITTFL complex corresponded to the distinct proportions of the effective ligament complex remaining on the fragment. The effective ligament complex served as the main ligament structure to maintain the rotational stability of the ankle. However, in the experiment, the ligament complex component on the fragment should be ensured to be not injured. In the preliminary experiment, the first set of models for maintaining the syndesmosis stability with only posterior malleolus fixation was preliminarily determined, and the corresponding posterior tibial insertion area of the ligament complex was 1/4S. Therefore, the four control models established in this study were 1/8S (model 1), 1/4S (model 2), 1/2S (model 3) and S (model 4). In addition, this study had important theoretical significance especially for the determination of the surgical indications of posterior malleolus fracture involving small articular surface area. Thus, in this model, the area ratio of the posterior malleolus involving the distal tibial articular surface was set to 1/5 (less than 25%). Based on the osteotomy of Macko et al.[9], the method of posterior malleolus osteotomy involving 1/5 articular surface was implemented and improved. The posteromedial point of the osteotomy line was changed to the most medial point of the tibial insertion of the PITFL and ITTFL complex, which was deemed by the authors to be more consistent with the actual situation.

### Analysis of Surgical Indications for Posterior Malleolus Fracture Based on the Rotational Stability of the Ankle

On the basis of the proposed osteotomy model, the surgical indications for posterior malleolus fracture were further determined based on the rotational stability of the ankle. Biomechanical analysis showed that, for the posterior malleolus fracture, the area threshold SF of posterior tibial insertion was 1/4S (Table 2). Combined with the difference in the antirotating ability (stiffness) between the two fixation methods, the posterior tibial area threshold, and the syndesmosis stability, the recommended surgical indications for posterior malleolus fracture are shown in Table 4. As previously mentioned, the influence of the posterior malleolus fracture on the stability of the ankle includes the posterior axial stability and posterolateral rotational stability. The ideal surgical indications for the posterior malleolus fracture should simultaneously restore the posterior axial stability and posterolateral rotational stability of the ankle, and the involved articular surface area and the displacement degree of posterior malleolus fragment should also be considered. Simple reduction and fixation of the posterior malleolus fracture was recommended when the syndesmosis was unstable, and the area ratio of posterior tibial insertion of posterior malleolus fracture was greater than or equal to 1/4. Simple posterior malleolus fixation achieved the dual effects of restoring and maintaining the rotational stability of the ankle and the axial stability of the tibiotalar joint, and the syndesmotic fixation was not necessary. Simple posterior malleolus fixation was not enough to maintain the rotational stability of the syndesmosis, and syndesmotic fixation should be performed when the syndesmosis was unstable and the area ratio was less than 1/4. After the syndesmotic fixation, the impacts of the posterior malleolus fixation indications on the rotational stability of the ankle need not be considered, and these indications only depended on the impact on the posterior axial stability, the involved articular surface area, and the displacement degree of posterior malleolus fragment. Therefore, the surgical indications for posterior malleolus fracture should still be combined with those in the literature and were regarded as necessary supplements to the classic surgical indications. When the syndesmosis was stable, regardless of the involved posterior tibial area ratio, the surgical indications only depended on the impact of the posterior malleolus fracture on the axial stability of the tibiotalar joint, the involved articular surface area, and the displacement degree of posterior malleolus fragment. In this case, the syndesmosis was already stable, so the influence of the posterior malleolus fixation on the rotational stability of the ankle need not be considered. In addition, further summary showed that syndesmotic fixation was only necessary when the syndesmosis was unstable and the involved posterior tibial area ratio was less than 1/4. In fact, the surgical indications for posterior malleolus fracture based on the axial and rotational stabilities of the ankle were complementary to each other. Especially, the surgical indications based on the rotational stability of the ankle had important theoretical significance for posterior malleolus fractures involving a small articular surface area.

### Quantitative Evaluation of the Influence of Posterior Malleolus Fracture and Fixation on the Rotational Stability of the Ankle

Another important contribution of this study is its quantitative assessment of the restoration of the rotational stability of the ankle with different fixation methods. Many studies have reported that the reduction and fixation of posterior malleolus fracture provide better rotational stability and reduce the incidence of syndesmosis instability and need for trans-syndesmotic screw [12, 16]. However, the specific posterior malleolus fracture type, in which simple posterior malleolus fixation was enough to restore and maintain the rotational stability of the syndesmosis and syndesmotic fixation was completely avoided, was not clearly defined. In clinical practice, the following situation often appears in cases of posterior malleolus fracture with syndesmotic diastasis: intraoperative stress test was positive initially, and the syndesmosis stability was restored following reduction and fixation of the posterior malleolus fracture in most cases. However, in a small number of cases, the stress test was still positive and further syndesmotic fixation was required. For such cases, the fixation method to restore the syndesmosis stability before or during the operation cannot be accurately preassessed. However, in this study, if the syndesmosis was unstable, simple reduction and fixation of the posterior malleolus fracture was enough to restore and maintain the rotation stability of the ankle when the area ratio of posterior tibial insertion of posterior malleolus fracture was greater than or equal to 1/4, and the syndesmotic fixation was recommended when the area ratio was less than 1/4. This conclusion provided a quantitative evaluation of the fixation methods for the clinical restoration of the rotational stability of the ankle and had positive theoretical significance.

### The Defects and Deficiencies

The main defects and deficiencies of this study were the limited number of fresh cadaver specimens and insufficient database, and it should be further supplemented and improved in the future.

## Conclusions

In conclusion, the surgical indications for posterior malleolus fracture have long been controversial. This study provides quantitative evaluation of the influence of posterior malleolus fracture and fixation on the rotational stability of the ankle, and promotes the supplement and improvement of the surgical indications for posterior malleolus fracture. Especially, the results have important theoretical significance for posterior malleolus fractures involving small articular surface area.

## Data Availability

The data sets supporting the conclusion of this article are included in the manuscript. Upon request, raw data can be provided by the corresponding author.

## Abbreviations

PITFL: Posterior inferior tibiofibular ligament
ITTFL: Inferior transverse tibiofibular ligament
